# Maize leaves drought-responsive genes revealed by comparative transcriptome of two cultivars during the filling stage

**DOI:** 10.1371/journal.pone.0223786

**Published:** 2019-10-30

**Authors:** Hongyu Jin, Songtao Liu, Tinashe Zenda, Xuan Wang, Guo Liu, Huijun Duan

**Affiliations:** 1 Department of Crop Genetics and Breeding, College of Agronomy, Hebei Agricultural University, Baoding, China; 2 North China Key Laboratory for Crop Germplasm Resources of the Education Ministry, Hebei Agricultural University, Baoding, China; National Institute of Plant Genome Research, INDIA

## Abstract

Like other important cereal crop in modern agricultural production, maize is also threatened by drought. And the drought stress during maize filling stage will directly affect the quality (protein or oil concentration) and also the weight of grain. Therefore, different from previous studies focusing on inbred lines and pot experiment at seedling stage, current study selected filling stage of the adult plant and planting maize in the experimental field. Two hybrids cultivars with different drought tolerant were used for drought and water treatment respectively. We performed transcriptome sequencing analysis of 4 groups, 12 samples, and obtained 651.08 million raw reads. Then the data were further processed by mapping to a reference genome, GO annotation, enrichment analysis and so on. Among them we focus on the different change trends of water treatment and drought treatment, and the different responses of two drought-tolerant cultivars to drought treatment. Through the analysis, several transcripts which encode nitrogen metabolic, protein phosphorylation, MYB,AP2/ERF, HB transcriptional factor, O-glycosyl hydrolases and organic acid metabolic process were implicated with maize drought stress. Our data will offer insights of the identification of genes involved in maize drought stress tolerance, which provides a theoretical basis for maize drought resistance breeding.

## Introduction

As the extremely important cereal crop in the world, Maize (*Zea mays* L.) is widely used in food, feed, and biofuel production for both humans and animals [1–3]. Like other crops, maize production is threatened by drought [4], which increases in severity, duration and frequency as climate changes, greatly reducing soil water available for plant uptake [5]. Consequently, drought and soil water deficit remains the main abiotic constraint to maize growth and production during the next few decades [6–8]. While maize production is affected by drought stress at all main growth stages, such as the seedling stage, flowering period and grain filling stage [9]. The extent of yield reduction from water deficits in maize depends on the growth stage at which the water deficiency happens, the severity and duration of the deficiency [10]. Substantial yield losses arise when drought occurs during grain filling stage [10].

Grain filling stage is the last growth stage in cereals [11], and the effects of water deficit on maize grain filling were also complicated. In general, drought increased the protein concentration of grains but decreased the oil concentration [10]. From previous studies, grain filling stage were shortened under drought conditions and with increased endogenous ABA levels due to water deficit, and the grain filling rate were accelerated. But, theoretically, shortening the grain filling stage was still unfavorable to maize grain weight [12].

Because maize has a facile model, large collection of mutant germplasm, presence of annotated genes and pathways, it not only has its economic and biological significance, but also a model plant of regulatory networks for studies [13]. Although tremendous progress has been made in the research of maize drought stress response mechanisms [14], yet due to technical reasons, previous studies have focused more on physiological and biochemical or single factor [15–18]. However, in this decade, with the development of rapid and economical sequencing technology and the completion of the maize B73 self-bred line genome sequence, the high throughput sequencing methods that are based on the RNA level and their research achievements have changed our view of the extent and complexity of maize transcriptome at a large extent [19]. Despite this, bunch of transcriptomics studies have focused on inbred lines as well as pot experiment at seedling stage [9]. Our perception of maize drought stress response mechanisms and genes involved still need to improve.

As the most successful crop that utilizes heterosis in modern agricultural production, maize hybrids are widely used in agricultural production instead of homozygous inbred lines [20]. Compared with inbred line, hybrid plant is stronger. In the same field conditions, the growth stages are not completely synchronous. Similarly, the widely studied drought resistance mechanism of maize at seedling stage is not necessarily the same as which at adult plant stage. Therefore, in this study, maize hybrid cultivar ND476 and ZX978 were selected as the research material to examine the molecular genetic mechanism of drought responsive during grain filling stage in maize. We performed comparative transcriptome analysis of these two hybrid cultivar’s leaves, with and without water, using Illumina HiSeq4000 sequencing platform to identify that the DEGs play an important role in maize. Our findings have promoted our understanding of the drought response mechanism of maize and provided a theoretical basis for breeding new drought resistant maize cultivars.

## Results

### Phenotypic responses of two maize cultivars to drought stress

**Fig 1:**
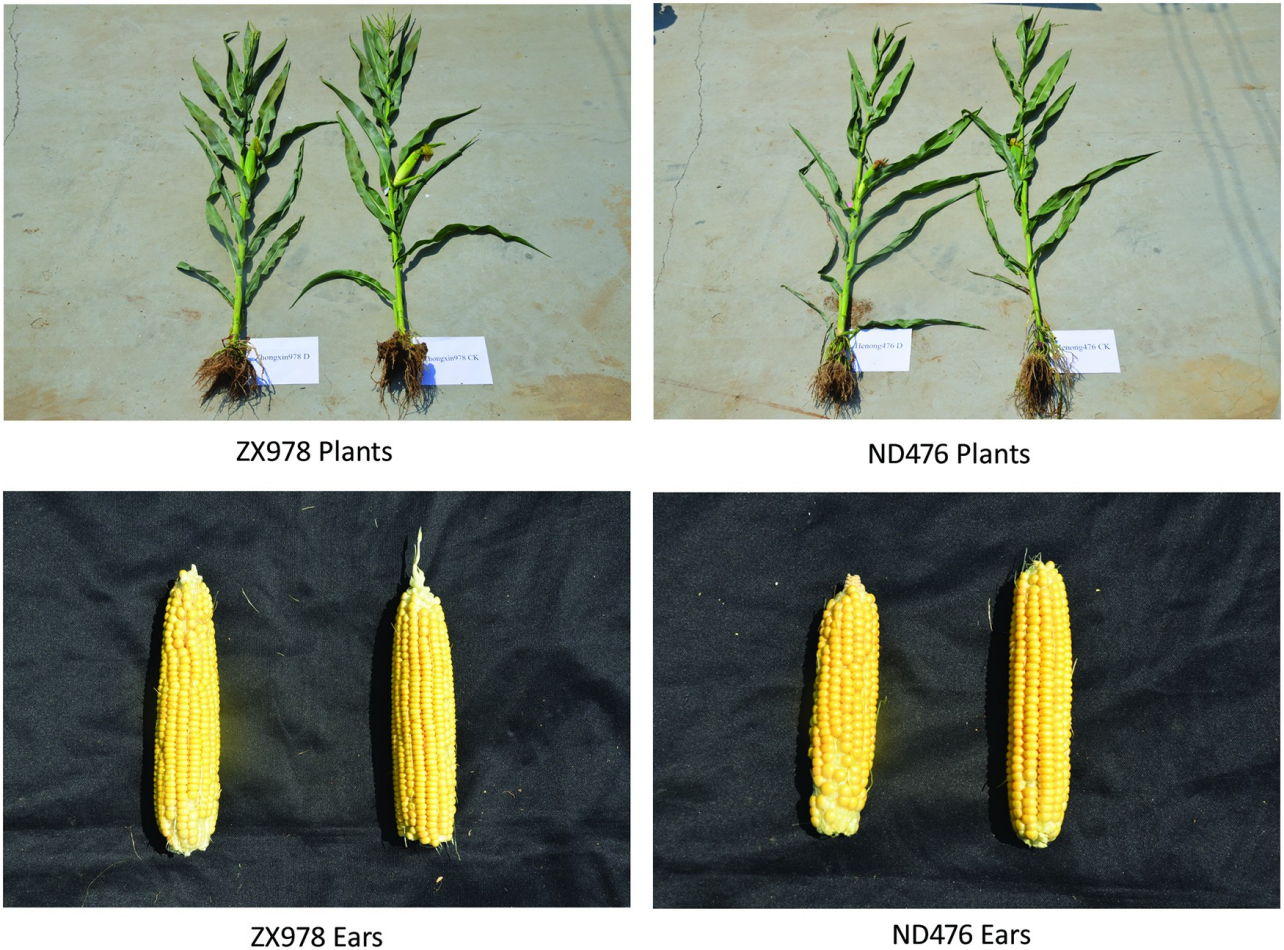
Two maize cultivars of ears and plants after drought and water treatment respectively, on the left is drought treatment, on the right is the water treatment.

Except for different water treatments or drought treatments, the two maize cultivars have grown under the same conditions for 14 days. As we can see in the photo (Fig 1), there is a significant difference in plant height between two treatments, also in both cultivars; plants under drought treatment are lower than the water treatment. In terms of leaves, drought treated plants showed shriveled up in both cultivars. But the difference of ears between the two cultivars was not obvious under the two treatments.

### RNA-sequencing and data processing

To obtain a maize gene expression profile, 12 cDNA libraries were constructed for deep sequencing using the Illumina High-Seq 4000 platform. These 12 samples categorized into four groups named as TC, SC, TD, and SD. Three samples for ND476 by treatment with water were named group TC. Three samples for ZX978 by treatment with water were named group SC. Three samples for ND476 by treatment without water were named group TD. Three samples for ZX978 by treatment without water were named group SD. After filtering, we obtained 646.74 million clean reads (Table 1), with 53.89 million reads on average from each sample. All sample’s minimum value of clean Q30 base rates, a key parameter that represents the quality of sequenced bases, were also greater than 96%. These reads were mapped by the use of Hisat2 and mapping rates float above and below 90%. The raw data were deposited in the NCBI Sequence Read Archive (SRA), under accession numbersSRP224988. Correlation analysis was performed on 3 sample replicates from each group. The results (S1 Table) showed that different replicates from each group are good.

**Table 1:** Number of reads sequenced and mapped to the maize genome.

Group	Sample	Raw reads	Raw bases	Clean reads	Clean bases	Q20(%)	Q30(%)	Total mapped	Uniquely mapped
TC	NDC1	48035096	7253299496	47610098	7121781293	98.92	96.33	43143631(90.62%)	41372616(86.9%)
	NDC2	54486068	8227396268	54166538	8115186558	99	96.56	50258591(92.79%)	48336953(89.24%)
	NDC3	44542680	6725944680	44085504	6589805593	98.97	96.5	38332401(86.95%)	36372979(82.51%)
TD	NDD1	57502078	8682813778	57188372	8564502124	99.02	96.62	53180180(92.99%)	51087820(89.33%)
	NDD2	60217732	9092877532	59870224	8967789116	98.97	96.48	55618716(92.9%)	53420048(89.23%)
	NDD3	54959348	8298861548	54580992	8178310681	99	96.56	49427404(90.56%)	47440596(86.92%)
SC	ZXC1	56850870	8584481370	56506150	8460982813	99.03	96.65	51191380(90.59%)	49427073(87.47%)
	ZXC2	52118476	7869889876	51767644	7756619879	99.02	96.63	46450490(89.73%)	44755259(86.45%)
	ZXC3	55508876	8381840276	55162792	8257374897	98.95	96.4	50381514(91.33%)	48418163(87.77%)
SD	ZXD1	55944494	8447618594	55592308	8325795684	98.96	96.42	50819133(91.41%)	48839828(87.85%)
	ZXD2	53660530	8102740030	53330596	7988769663	98.98	96.49	48023453(90.05%)	46046314(86.34%)
	ZXD3	57250354	8644803454	56881498	8517462308	98.96	96.44	51900338(91.24%)	49700388(87.38%)

Noted: NDC1, NDC2, NDC3 are 3 samples of hybrid cultivar ND476 by treatment with water. NDD1, NDD2, NDD3 are 3 samples of hybrid cultivar ND476 by treatment without water. And ZXC1, ZXC2, ZXC3 are 3 samples of hybrid cultivar ZX978 by treatment with water. ZXD1, ZXD2, ZXD3 are 3 samples of hybrid cultivar ZX978 by treatment without water.

### Transcriptomic responses

**Fig 2:**
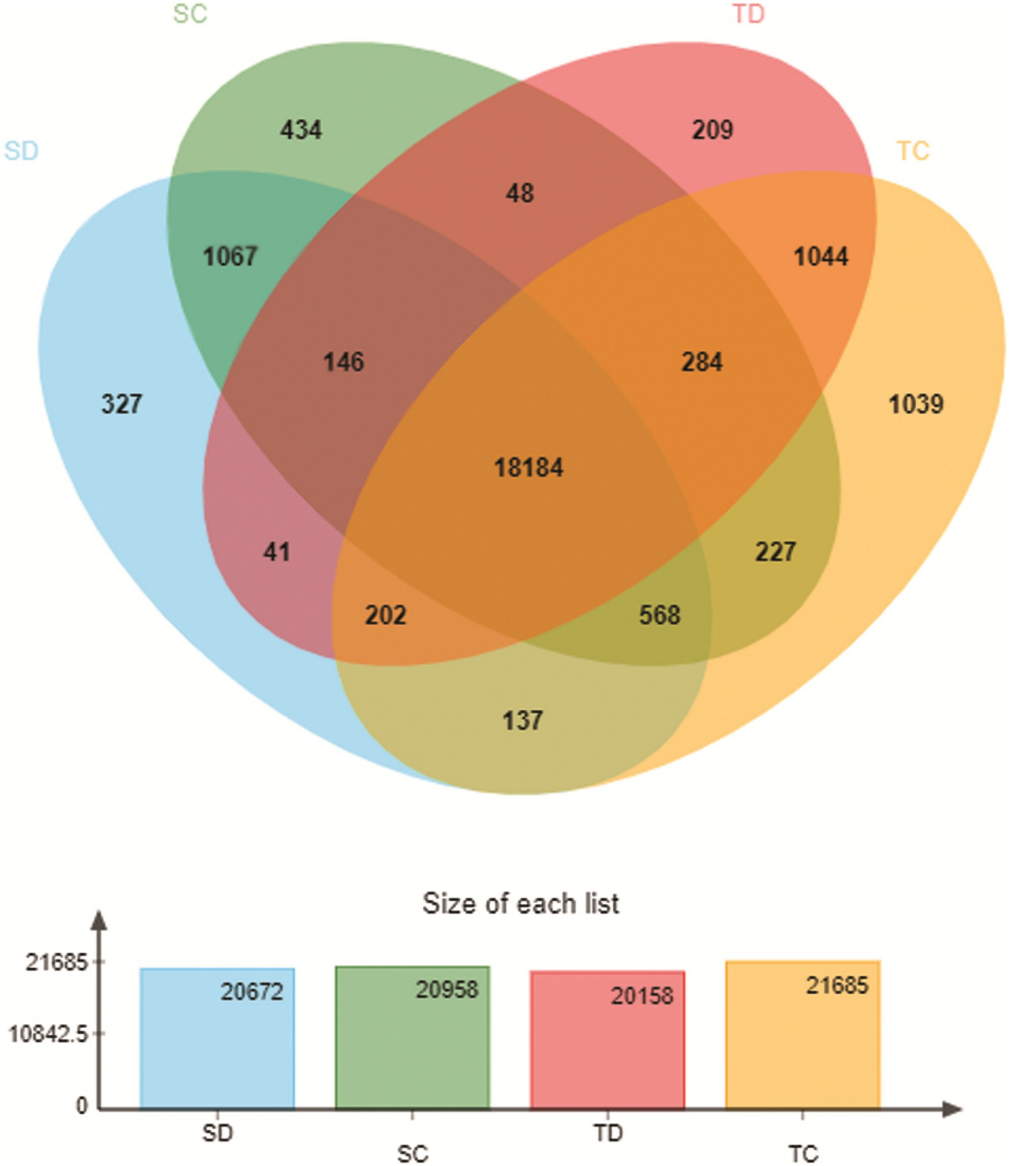
The expression numbers of transcriptomic responses in each treatment list by using a Venn diagram.

Gene expression levels were calculated in the fragments per kilobase of transcript per million fragments mapped (FPKM) by using RSEM (http://deweylab.github.io/RSEM/). By using an FPKM value ≥ 1 as a criterion, we detected at least 20,000 genes in each group. The number of expressed genes is shown in Venn diagram (Fig 2). The sensitive cultivar (ZX978) expressed 20672 and 20958 genes under drought stress condition (SD) and normal condition (SC), respectively. The tolerant cultivar (ND476) expressed 20158 and 21685 genes under drought stress condition (TD) and normal condition (TC), respectively. The number of genes expressed in the two cultivars under drought treatment was less than which under normal condition. The expressed rates of 18184 genes shared between each treatment ranged from 83.86% to 90.21%.

209 genes were exclusively expressed in tolerant cultivar ND476 with drought treatment (TD). GO enrichment analysis was performed on this group by using Goatools software (Fisher test, Padjust<0.05). The results showed that GO:0033609 (oxalate metabolic process) and GO:0009834 (plant-type secondary cell wall biogenesis) were the most significantly enriched GO terms in the biological process (BP) category. Within the molecular function (MF) category, GO:0045735 (nutrient reservoir activity), GO:0019863 (IgE binding) and GO:0019865 (immunoglobulin binding) were most significantly enriched.

### Down or up regulated genes after drought treatment

**Fig 3:**
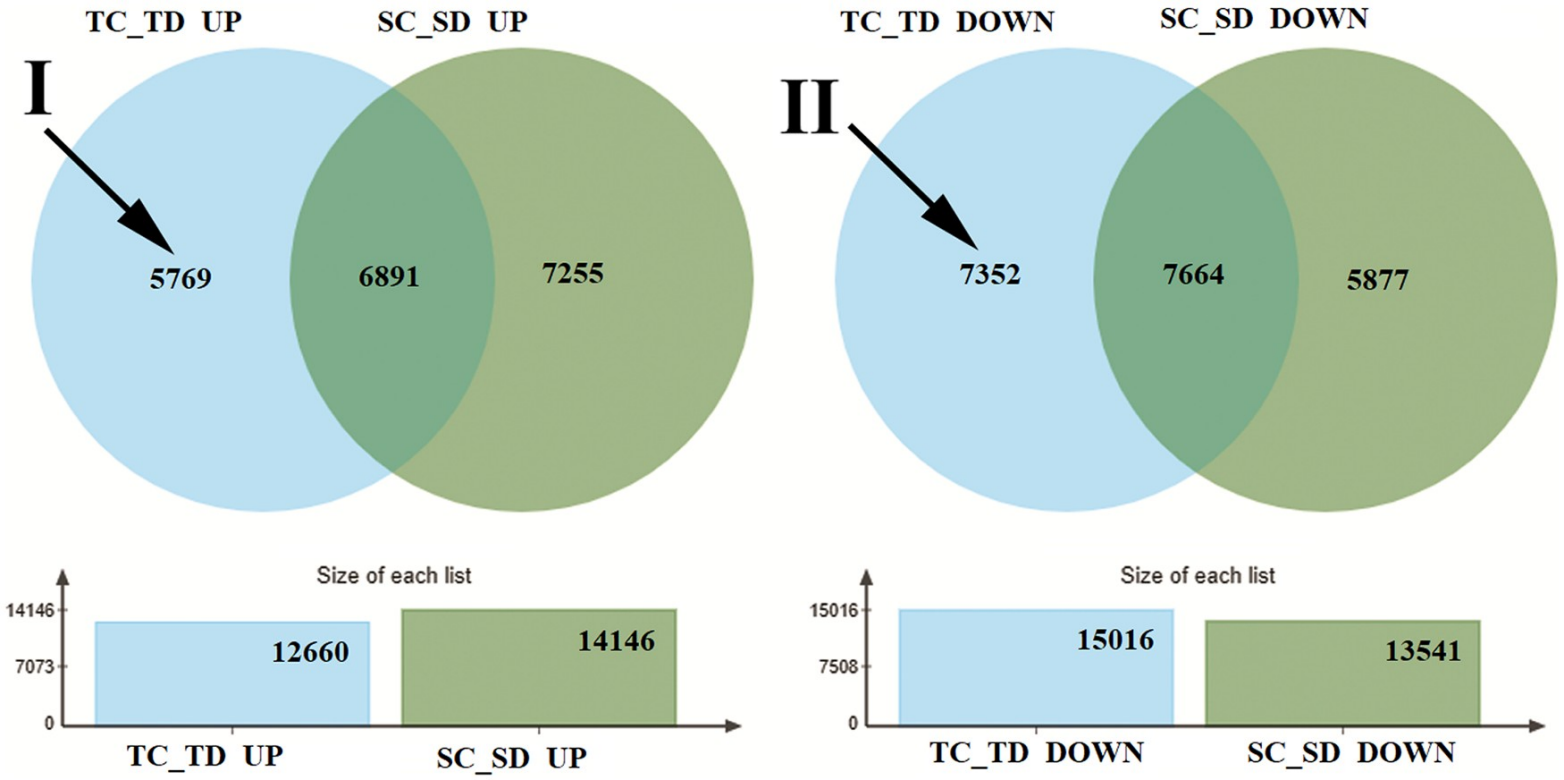
The number of up or down regulated genes between water treatment and drought treatment in two cultivars by using a Venn diagram. Area I: 5769 up regulated genes only expressed in TC_TD. Area II: 7352 down regulated genes only expressed in TC_TD.

**Fig 4:**
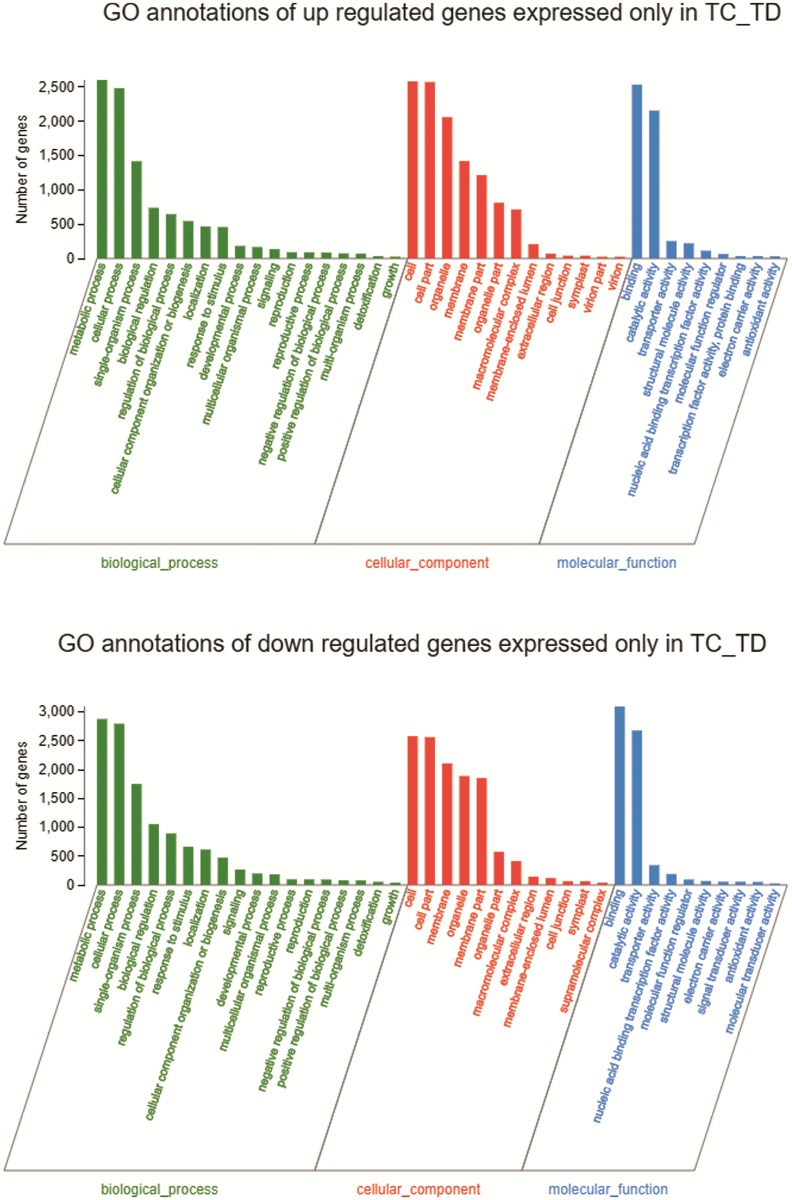
GO annotations and categories of down or up regulated genes expressed only in TC_TD. GO analysis of Area I and Area II.

To determine those genes that control response to drought stress, we compared the mRNA expression difference with and without water in two cultivars(SC_SD and TC_TD) by using DESeq2 software. Considering the constitutive expression of ZX978 and ND476 cultivars and their response to the same physiological cycle (Grain Filling Stage) and environment, we tried to reduce the influence of background on gene expression by using Venn’s analysis (Fig 3) to find genes that are more related to drought resistance. Through Venn’s analysis of the differential expression result, we found that a total of 19915 upregulated genes, composed of 5769 genes only in TC_TD (Area I, in Fig 3), 7255 genes only in SC_SD and 6891 genes in both TC_TD and SC_SD. On the other hand, 20893 genes were downregulated, composed of 7352 genes only in TC_TD (Area II, in Fig 3), 7664 genes only in SC_SD and 5877 genes in both TC_TD and SC_SD. Then we GO annotated the up (Area I) or down (Area II) regulated genes in the unique areas of TC_TD (Fig 4).

Through the GO enrichment analysis of 5769 genes up-regulated only in TC_TD (S2 Table, Area I), we can find that GO:0022618 (ribonucleoprotein complex assembly), GO:0009657 (plastid organization), GO:0071826 (ribonucleoprotein complex subunit organization) and GO:0065003 (macromolecular complex assembly) were common and the most significantly enriched GO terms in the biological process (BP). Within the molecular function (MF) group GO:0005198 (structural molecule activity) and GO:0003735 (structural constituent of ribosome) were most enriched. On the other hand, the GO enrichment analysis of 7352 genes down-regulated only in TC_TD (S3 Table, Area II) shown that GO:0006468 (protein phosphorylation), GO:0016310 (phosphorylation) and GO:0006793 (phosphorus metabolic process) were most significantly enriched GO terms in the biological process (BP).

### Identification and functional categorization of the DEGs in ND476 between ZX978 samples

**Fig 5:**
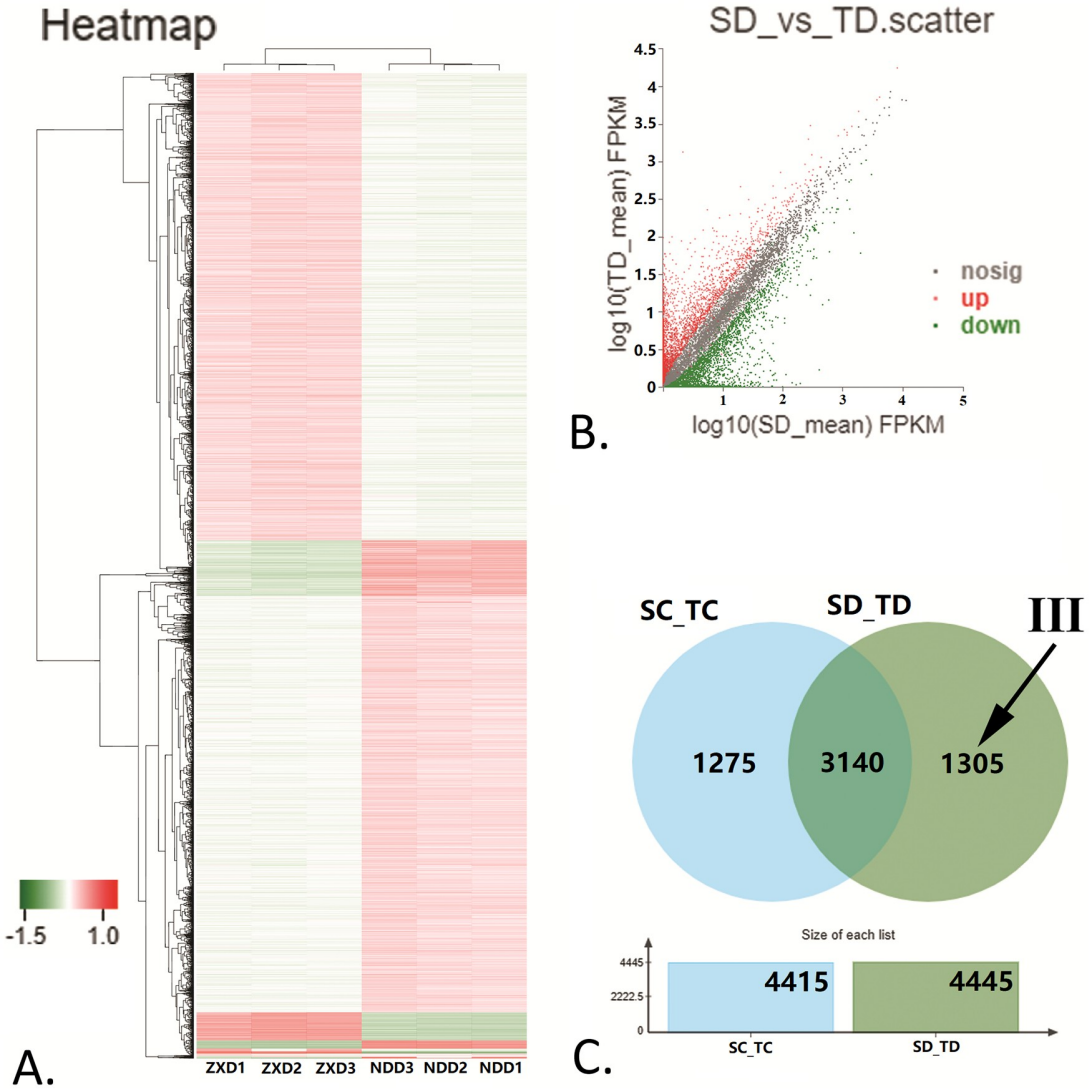
Analysis of differentially expressed genes (DEGs) between ZX978 and ND476. (A.) Heat map showing clustering analysis of DEGs between the two cultivars after drought stress treatment (SD_TD). (B.) Scatter plot showing the (log10 FPKM) expression of the DEGs in the SD_TD. (C.) Venn diagram showing the number of DEGs between SC_TC and SD_TD. Area III: 1305 DEGs only expressed in SD_TD.

Through DESeq2 software, using p-adjust<0.05, FC>2 or FC < 0.5 as the measurement standard, we identified differentially expressed genes (DEGs). Under water treatment, we identified 4415 (2447 up and 1968 down regulated) DEGs between the tolerant and sensitive cultivars (SC_TC). Another side, under water-deficit conditions, 4445 (2190 up and 2255 down regulated) DEGs were observed between the tolerant and sensitive cultivars (SD_TD) which illustrated in the scatter plot (Fig 5B) that shows the distribution of DEGs. For further understanding of the differential gene expression patterns between maize sensitive cultivar ZX978 and tolerant cultivar ND476, we conducted hierarchical clustering based on gene expression patterns. As heat map (Fig 5A) shown, in drought stress treatment, DEGs were grouped into several clusters, and more DEGs were down regulated than up regulated.

**Fig 6:**
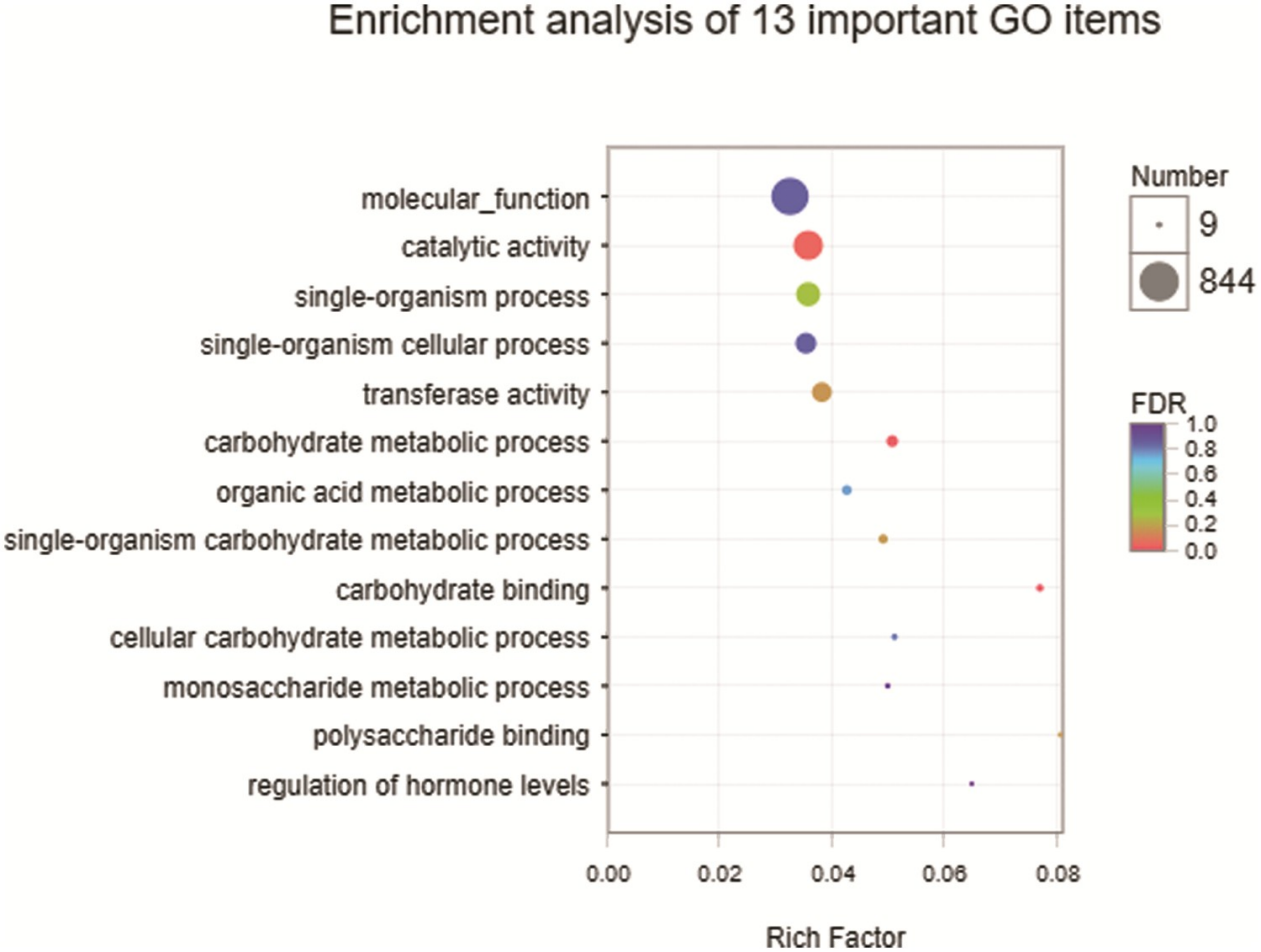
GO enrichment of 13 important terms. The “rich factor” shows the ratio of the number of the DEGs to the total gene number (Background number) in certain GO terms.

Venn analysis (Fig 5C) was performed on the two gene sets to obtain area (Area III, in Fig 5C) more closely related to drought stress resistance, and it contains 1305 DEGs (29.36% of SD_TD) which is also probably to be the cause of significant difference in drought dress resistance between sensitive and tolerant cultivar. As in area SD_TD, there are more down regulated DEGs (746, 57.16%) than up regulated DEGs (559,42.84%) in this area. All the DEGs got GO annotation and enrichment analysis. The common DEGs to both cultivars were mainly involved in metabolic process, catalytic activity, binding and cellular process. The 1305 DEGs (P-value< 0.05) in SD_TD’s unique area (Area III) were enriched in 233 terms, of which 134 were biological process (BP), 18 were cellular component (CC) and 81 were molecular function (MF). The five most highly enriched GO terms in MF and BP were found (Fig 6) to be GO:0003674 (molecular_function), GO:0003824 (catalytic activity), GO:0044699 (single-organism process), GO:0044763 (single-organism cellular process) and GO:0016740 (transferase activity).

### DEGs encoded transcription factors in tolerant cultivar ND476's unique area

77 DEGs were expressed in Tolerant Cultivar ND476 (Area III) which encoded Transcription Factors (TFs), corresponding to 256 transcripts (137 down, 119 up). The main TF families (Table 2, S4 Table) included were AP2/ERF, HB, MYB_related, NAC and bHLH (42 DEGs, 54.55%). In the high expression frequency DEGs, *Zm00001d033553* (GRMZM2G068476_P01) was up regulated, which is Zinc finger CCCH domain-containing protein 33, connected with metal ion binding and belonging to the C3H TF family. *Zm00001d021442* (GRMZM2G053298_P01) is NLP-transcription factor 13, down regulated. *Zm00001d031061* (GRMZM2G003509_P01) was also down regulated, which is Homeobox-transcription factor 119 and connected with lipid binding and DNA binding. There are 5 different transcripts encoding it, and they belong to the HD-ZIP and HB TF families respectively. No significant up or down trend was observed in transcription factors.

**Table 2:** Family table of transcription factors.

TF_family	Gene Number	Transcript Number
AP2/ERF	10	12
B3 superfamily	1	1
C2C2	6	13
C3H	2	45
CO-like	1	2
DBB	1	2
GRAS	1	2
GRF	2	3
HB	10	24
HB-PHD	1	2
HD-ZIP	1	5
HSF	1	2
LBD (AS2/LOB)	2	5
MADS	2	4
MIKC	1	4
MYB	3	6
MYB_related	10	30
NAC	7	16
Nin-like	1	18
TALE	1	3
TCP	3	6
WRKY	6	12
ZF-HD	1	1
bHLH	7	14
bZIP	3	5

### KEGG annotation and analysis of DEGs in tolerant cultivar ND476's unique area

We further analyzed the biologic pathways involved in DEGs which expressed in tolerant cultivar ND476's unique area (Area III) by mapping them to the Kyoto Encyclopedia of Genes and Genomes (KEGG) database. Through the KEGG annotation of DEGs in Area III (S5 Table), we focused on the metabolism which included more than half of the annotated DEGs, in the level of first category. In the metabolism, carbohydrate metabolism has the largest number of annotated DEGs in the level of second category with a total of 52 DEGs, and with Amino acid metabolism (29), Lipid metabolism (23), Energy metabolism (22), Biosynthesis of other secondary metabolites (21), etc. Then in carbohydrate metabolism, the top three terms in KEGG pathways with most involved DEGs are: Starch and sucrose metabolism (map00500), Inositol phosphate metabolism (map00562), Glycolysis / Gluconeogenesis (map00010).

### Nodes of correlation of gene expression in tolerant cultivar ND476's DEGs

Based on the correlation of gene expression, spearman correlation algorithm was adopted to obtain the correlation coefficient between genes and the number of connections of each node (S6 Table). We know little about the first two DEGs (*Zm00001d001272* and *Zm00001d001602*) with the highest number of links, but both of those were expressed only in SD, which may be one of the reasons for the differences between the two cultivars. The third, *Zm00001d000094*, which is related to metabolic process (BP) and amino acid binding (MF) in GO annotation, also expressed more in SD than TD. The 1305 DEGs in SD_TD’s unique area (Area III), 116 key nodes with more than 100 links were counted, most of which (103) were down regulated. Even more, the number of links greater than 150 of the 30 key nodes is all down regulated.

### RT-qPCR validation of RNA-Seq data

**Fig 7:**
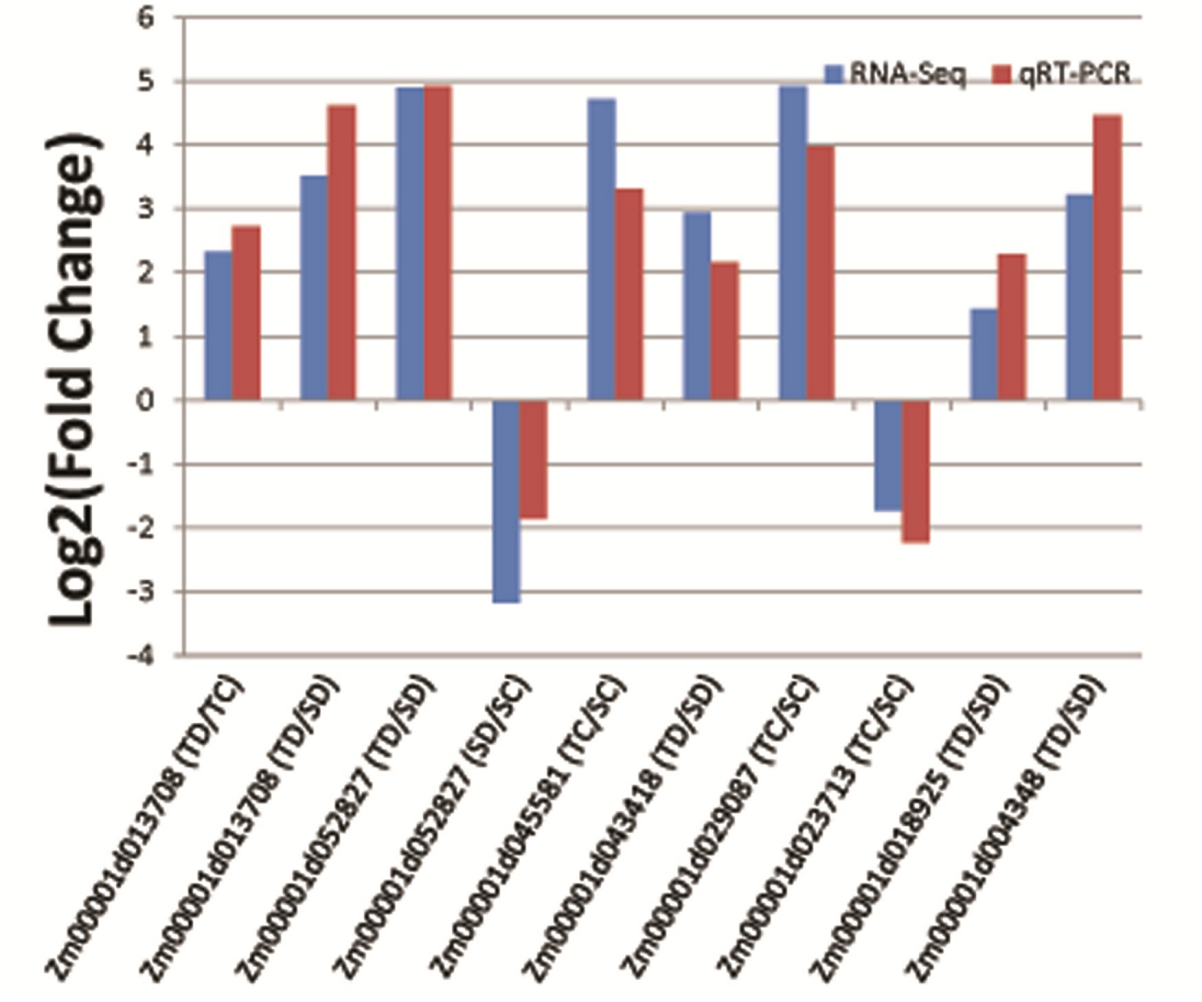
Validation results of RNA-Seq data using qRT-PCR. “TD/TC”, “TD/SD”, etc. represent the groups in which DEG is selected.

Base on the results of our functional annotation and analysis combined with the previously published research, 10 candidate genes (Table 3) were randomly selected for qRT-PCR analysis. The samples used were the same as those used for RNA sequences. The expression patterns of 10 genes in qRT-PCR and RNA-Seq were consistent (Fig 7). The R-squared were 0.87, which confirmed that the results of the RNA-Seq were reliable.

**Table 3:** List of qRT-PCR primers used in this study.

Gene ID	Gene Description	Sequence 5'to 3'		log2FC
Zm00001d013708	Probable prefoldin subunit 4	F:	CGAGAACCTTGACGATGCTG	2.7262
In TD/TC		R:	GATGGCGTCCTTGAACTTCC	
Zm00001d013708	Probable prefoldin subunit 4	F:	CGAGAACCTTGACGATGCTG	4.6084
In TD/SD		R:	GATGGCGTCCTTGAACTTCC	
Zm00001d052827	Salicylate/benzoate carboxyl methyltransferase	F:	AGGCAGTTCCAGGAGGACAT	4.9209
In TD/SD		R:	AATTAAGAGAAGGGACCTGTTTGAC	
Zm00001d052827	Salicylate/benzoate carboxyl methyltransferase	F:	AGGCAGTTCCAGGAGGACAT	-1.8564
In SD/SC		R:	AATTAAGAGAAGGGACCTGTTTGAC	
Zm00001d045581	Putative MYB DNA-binding domain superfamily protein	F:	TTTCCAGGTCCCAACTGCG	3.3139
In TC/SC		R:	GTCAGCGCCAGTGAGACAA	
Zm00001d043418	La-related protein 6A	F:	GGTTGTCCGATGCTTGTCAT	2.1635
In TD/SD		R:	GCCAAGTTGAACCTCACTTGTAG	
Zm00001d029087	Sucrose synthase 3	F:	CGATTAACACCGCCTGCTACT	3.9696
In TC/SC		R:	GGTGGGAACCTTGGTGCAT	
Zm00001d023713	Photosystem I reaction center subunit N	F:	GCAGAAGGTAGTTCGGTCTCA	-2.2342
In TC/SC		R:	GTCGCTGAGGAAGGGTACTT	
Zm00001d018925	Pumilio homolog 3	F:	GAGCTACCACAAGTTCGCCT	2.2872
In TD/SD		R:	AGCCACCACGTACTAACCAC	
Zm00001d004348	Natterin-4	F:	AAGTGTCCTCTACGCCTGGT	4.4616
In TD/SD		R:	TTTGTGACGGATCGGAGGGA	
GAPDH	Internal reference gene	F:	ACTGTGGATGTCTCGGTTGTTG	—
		R:	CCTCGGAAGCAGCCTTAATAGC	

## Discussion

As we know, plants have complex adaptation mechanisms at many different levels, and the molecular mechanisms supporting this phenomenon remained not entirely clear [21,22]. In the current report, we focused on specific area of the gene set to reduce the impact of different genetic backgrounds and thus obtain genes that are closely related to drought responsive.

### Differences between two cultivars treated with and without water in gene expression

After 15 days of water treatment and drought treatment, through transcriptome sequencing and differentially expressed genes analysis, we were surprised to find that only a small number of genes in the two cultivars reached the level of statistically significant difference in gene expression. This is in contrast with our expectations and previous studies which have reported a maize transcriptome analysis in drought response [23]. We thus hypothesize [24,25] that a drought period of 15 days may be not long enough to drive the full upregulation of the maize genome, In particular, maize itself is a drought-tolerant crop, and compared with the seedling stage selected in most experiments, the drought tolerance of maize in the mature stage is stronger, moreover, in this experiment, the DEGs in tolerant cultivar (two in TC_TD) were less than those in sensitive cultivar (nine in SC_SD).

We focus on up or down regulated of genes in the unique area of TD_TC (Area I and Area II) to search for the molecular mechanism of drought resistance in ND476 cultivars. As can be seen from the enrichment analysis of the unique area (Area I), at different GO levels, a bunch of biosynthetic related terms were up regulated. GO:0009059 (macromolecule biosynthetic process), GO:0044283 (small molecule biosynthetic process), GO:0043043 (peptide biosynthetic process), GO:0043604 (amide biosynthetic process) and GO:0008652 (cellular amino acid biosynthetic process) were both observed. In addition, GO:1901564 (organonitrogen compound metabolic process) and GO:0006518 (peptide metabolic process) were also up regulated. It’s similar to previous studies that modulating the expression of genes influencing nitrogen metabolic may be a cause of drought tolerance in maize [26].

After modification, proteins have new functions in binding, catalysis, regulation and physical properties. And protein phosphorylation, as one of the most common post-translational modifications, can transiently modify protein properties, like enzymatic activity, subcellular localization, protein structure and stability, and interactions with other proteins [27]. Moreover, protein phosphorylation, which controls stress responses by transmitting stress signals from the cell surface to the nucleus, is a universal biochemical signal in cells and a central post-translational modification (PTM) in ABA signaling [28,29]. In the current study, among the genes in the unique area of TD_TC (Area II), GO:0006468 (protein phosphorylation), GO:0016310 (phosphorylation), GO:0006793 (phosphorus metabolic process), GO:0006796 (phosphate-containing compound metabolic process) several biological process were down regulated. Within the molecular function (MF) category, GO:0005543 (phospholipid binding) and GO:0005544 (calcium-dependent phospholipid binding) were enriched, which have been identified that phospholipid signaling pathways play a key role in plant responses to environmental stresses by previous studies [30].

### Transcription factors that respond to drought stress

When plants are subjected to drought stress, transcription factors (TFs) promotes molecular response by activating or repressive the function of target genes [31]. A bunch of TF families, such as AP2, ERF, MYB, bZIP, bHLH, GRAS, WRKY, NAC, NF-YA, and NF-YB, have been shown to be involved in physiological regulation and molecular functions, like stomatal regulation, hormone signaling, root development and osmoregulation [32]. As in the current study, two genes, Zm00001d053311 (GRMZM2G065374_P01) and Zm00001d047017 (GRMZM2G049229_P01), which both belong to HLBL TF family, are involved in Plant hormone signal transduction. Except for NF-YA and NF-YB, all the above TFs had a considerable number of coding DEGs expressions in the SD_TD’s unique area (Area III) of this experiment. Maize proteins encoded by AP2/ERF TFs can regulate a multitude of transcriptional programs to potentially participate in a variety of stress responses [33]. HB family also has abundant coding DEGs, which have been reported in the study of carotenoids, a diverse group of colorful pigments, contribute to the development in plants [34]. The gene of MYB family in wheat overexpression experiment has shown that it positively regulates plant response to drought stress [35]. In our experiment, the expression of MYB family was also consistent with it. Five genes (four *Zm00001d041576* and one *Zm00001d032024*) in the SD_TD’s unique area (Area III) were up regulated, only one (*Zm00001d008528*) were down regulated. However, as mentioned above, no significant up or down trend was observed in most of TF families. Maybe several TF genes did not show any differential expression pattern in the selected tissues due to an environmental condition, genetic background effect and these TF genes interacting with each other in complex networks [36].

### Analysis on the difference of drought tolerance between two cultivars

As the main physiological functions underpinning growth and the core of bio-molecular metabolism, carbohydrate metabolism changes in the gene expression level is the adjustment and feedback of plants under drought stress [3, 37]. In the current study, we conducted enrichment analysis on 1305 DEGs of SD_TD’s unique area (Area III) and obtained GO:0030246 (carbohydrate binding) and GO:0005975 (carbohydrate metabolic process), which can be further classified as GO:0030247 (polysaccharide binding), GO:0044723 (single-organism carbohydrate metabolic process), GO:0044262 (cellular carbohydrate metabolic process) and GO:0005996 (monosaccharide metabolic process) at different GO levels. Among them, malate dehydrogenase was up regulated, which is one of the key enzymes in carbohydrate metabolism and can catalyze the reversible conversion between malic acid and oxaloacetic acid. It has been reported that the enzyme activity in maize inhibited after drought treatment, and the up regulation in this experiment may be a reason of its drought tolerant mechanism [38,39]. Many kinds of glycosyltransferase and glycosyl hydrolase were also observed, including O-Glycosyl hydrolases family 17 protein (*Zm00001d013097*, *Zm00001d018214*), UDP-glycosyltransferase 71B1 (*Zm00001d006449*) and UDP-glycosyltransferase 76C1 (*Zm00001d019256*), etc. O-glycosyl hydrolases are a large and diverse family of enzymes which hydrolyze the glycosidic bond, of which its response to biotic and abiotic stresses has been reported In Oryza sativa L. and Zea mays L. [39–41]. However, a family of O-glycosyl hydrolases in this experiment were both up and down regulated. Family of glycosyltransferases are related with improving the properties of secondary metabolites and have significantly enriched the chemical species. A member of family, UFGT2, has been reported involved in modifying flavonols, contributes to improving plant tolerance to abiotic stresses [42]. It’s similarly with us that all of four UDP-glycosyltransferases were up regulated. Endoglucanase correlated with cell-wall extensibility, which has been reported in maize under NaCl stress [43]. However, in this experiment, both genes (*Zm00001d015292*, *Zm00001d044744*) described as Endoglucanase 11 were up-regulated and down-regulated, which may mirror the complexity surrounding as plants battle drought stress. It is worth mentioning that Ribulose-bisphosphate carboxylase (rubisco), which plays an important role in carbon fixation, was not significantly differentially expressed in this experiment, but it was down-regulated in six of ten related genes in SC_SD and up-regulated in eight of ten genes in TC_TD.

Similarly, in our KEGG analysis of Area III, carbohydrate metabolism has the largest number of annotated DEGs. The importance of starch and sucrose metabolism (map00500) during maize early kernel development had been reported in previous studies [44]. This pathway term has 13 annotated DEGs in current study, and also suggested that drought has a considerable influence on maize grain filling.

Plant hormones are a variety of trace endogenous compounds. Ultra-trace amounts of plant hormones can play a vital role in plant growth, development and quick response to biotic and abiotic stresses, and the plant growth reduction under drought stress conditions could be an outcome of altered hormonal balance [45,46]. In our data, nine DEGs were enriched in GO:0010817 (regulation of hormone levels), including brassinosteroid synthesis1 (*Zm00001d028325*), brassinosteroid-deficient dwarf1 (*Zm00001d033180*), abscisic acid 8'-hydroxylase2 (*Zm00001d051554*) and abscisic acid 8'-hydroxylase3 (*Zm00001d050021*), etc., and all four of them were upregulated. As a plant steroid hormone, Brassinosteroid is a regulator of the plant drought response [47,48]. Brassinosteroid-deficient dwarf1 gene encodes a Brassinosteroid C-6 Oxidase that catalyzes the final steps of brassinosteroid synthesis [49]. Interestingly, it’s also associated with established height loci in maize [50]. Abscisic acid (ABA) is essential for drought tolerance, and plants with ABA overexpression showed better drought tolerance [51–53]. However, ABA 8'-hydroxylase hydroxylates ABA to 8'-hydroxy- abscisate and NADP+ thereby depleting ABA to reduce ABA levels and produced ABA-deficient phenotypes [51, 54].

As an important part in the plant adapting to adversity stress, organic acid metabolic process has been reported under alkali, salt and aluminum stress [55,56]. In our study, 60 DEGs were enriched in GO:0006082 (organic acid metabolic process), of which Tyrosine decarboxylase 1 (*Zm00001d024664*) and Tyrosine aminotransferase (*Zm00001d016441*) were both down regulated, meanwhile Tyrosine—tRNA ligase 1 cytoplasmic (*Zm00001d049579*) were up regulated, which would help improve the accumulation of tyrosine. And the accumulation of organic solutes including amino acids help plants to overcome drought stress through osmotic adjustment [57]. The precursor of 18C unsaturated fatty acids, which account for more than 70% of the fatty acids in plant membrane lipids, is produced by stearoyl-acyl-carrier-protein desaturase (SACPD) [58]. SACPD are involved in plant defense responses including cold or high temperature stress, and has been reported in arabidopsis, nicotiana and lima bean [58–60]. However, the two related genes stearoyl-acyl-carrier-protein desaturase2 (*Zm00001d012033*) and stearoyl-acyl-carrier-protein desaturase10 (*Zm00001d024273*) were both down regulated in our data, which may not be the main drought response mechanism in our plant.

## Conclusions

In this study, we have compared the leaf transcriptome responses of drought-tolerant ND476 and drought-sensitive ZX978 maize hybrid cultivar after 15 days drought treatment, during the maize filing stage, including 4 groups, 12 samples. By using an RNA-seq approach, we focus on the different change trends of water treatment and drought treatment in two cultivars, and also 1305 DEGs which are expressed only in tolerant cultivar ND476 with drought treatment. Our analysis revealed that the drought-resistance in maize is related to the regulated expression of some key genes, such as biosynthetic related, nitrogen metabolic process, phospholipid signaling pathways, MYB,AP2/ERF, HB transcriptional factor, malate dehydrogenase, O-glycosyl hydrolases, plant hormones and organic acid metabolic process. Our findings provide useful reference data for maize drought stress response mechanisms during filling stage, and also provide a theoretical basis for maize drought resistance breeding.

## Materials and methods

### Plant materials and drought stress treatment

Two maize (*Zea mays* L.) hybrids cultivars with contrasting drought sensitivity were used in this experiment (tolerant ND476 and sensitive ZX978). Seeds were provided by the North China Key Laboratory for Crop Germplasm Resources of Education Ministry, Hebei Agricultural University, China. The field experiment was carried out in the drought-resistance canopy at Qingyuan Experimental Station (115°E, 38°N) in 2018. The canopy of the station was opened on non-rainy day, and could be automatically closed on rainy days to enable continuous drought. The two cultivars were sowed in four blocks, two for water treatment and two for drought treatment. For both tolerant and sensitive cultivars, a half of the maize stop watering completely and the other maize were grown under well-watered condition. We started the drought treatment, about 45 days after germination, on the 11th of August and continued the artificial drought for 12 days until the afternoon of the 23th of August. Flag leaves collected from the control and drought stress treated maize after 12 days treatment, each had three technical replicates, for both RNA-Seq and qRT-PCR experiment, then frozen in liquid nitrogen immediately, and stored at −80 ◦C.

### Library construction, RNA sequencing and data process

Total RNA was extracted from the flag leaves samples using Plant RNA Purification Reagent base on the manufacturer’s instructions (Invitrogen) and genomic DNA was removed using DNase I (TaKara). The concentration and purity of RNA were detected by Nanodrop2000. The integrity of RNA was detected by agarose gel electrophoresis, and RIN value was determined by Agilent2100. For passed samples, RNA-seq transcriptome libraries were prepared by using TruSeqTM RNA sample preparation Kit from Illumina (San Diego, CA). At last, sequencing were conducted on an Illumina Hiseq4000 platform in Shanghai Majorbio Bio-pharm Technology Co.,Ltd. Raw sequencing reads were quality controlled by using SeqPrep software (https://github.com/jstjohn/SeqPrep) and Sickle software(https://github.com/najoshi/sickle). Then clean reads were separately mapped to the maize reference genome with orientation mode by using TopHat2 software (http://ccb.jhu.edu/software/tophat/index.shtml).

### Gene annotation and DEG’s GO enrichment

For functional annotation, we annotated all the genes using the GO database. GO is the genomic ontology consortium’s comprehensive database of all gene-related research across the world. Gene expression levels were calculated with FPKM as the standard by using RSEM software. The DEGs of several important gene sets were identified by using DESeq2 software. And the DEGs from these important areas were enrichment analyzed by using Goatools software. Similarly, we also annotated part of DEGs we interested by mapping them to the Kyoto Encyclopedia of Genes and Genomes (KEGG) database. In addition, we also identified the TFs in important gene sets, PlantTFDB 4.0 was used as the database, Hmmscan software was used to analyze the transcription factor family of plant-derived genes, and transcription factor annotation was conducted through Blast.

### Quantitative real time-PCR (qRT-PCR) analysis

Quantitative real-time PCR (qRT-PCR) was performed to confirm the results of RNA sequences. We randomly selected 10 candidate genes, and *GAPDH* gene was also selected as internal reference gene, and gene specific primers were designed by using PRIMER 3 software in Shanghai Wcgene Biotech Co.,Ltd. For first-strand cDNA synthesis, 2 μl of RNA was reverse-transcribed into 20 μl cDNA by using HiFiScript cDNA Synthesis Kit (CoWin Biosciences). The program was performed with 20 μl total reaction volume, including 10 μl of Super EvaGreen Master Mix (US EVERBRIGHT INC), 0.5 μl of each forward primer (50 pmol) and reverse primer (50 pmol), 1 μl of template cDNA, and each sample had three technical replicates (Z’s paper). The results were processed by 2−ΔΔCT method to calculate the relative mRNA abundance [61].

## Supporting information

S1 Table The Spearman correlation of different samples based on FPKM values. (XLSX)

S2 Table GO enrichment analysis of 5769 genes up-regulated only in TC_TD. (XLS)

S3 Table GO enrichment analysis of 7352 genes down-regulated only in TC_TD. (XLS)

S4 Table Frequency of transcription factor expression in tolerant cultivar ND476's unique area. (XLSX)

S5 Table KEGG pathway annotation and class for Area III. (XLS)

S6 Table Number of connections of each gene node. (XLS)
